# Severe pneumococcal pneumonia with human rhinovirus/enterovirus coinfection leading to sepsis in a welder: a case report

**DOI:** 10.3389/fmed.2026.1864223

**Published:** 2026-07-13

**Authors:** Pavlina Peneva, Greta Kossian, Leon Nedelcev, Richard Coveney, Pavel Yordanov, Valentina Dimitrova, Petar Georgiev, Nikoleta Mircheva, Figen Mustafa, Anton Antonov, Miglena Tzenova, Antoanina Botseva, Nadezhda Ilieva, Velislava Tasheva, Yulia Doga, Diana Petkova, Tanya Dobreva

**Affiliations:** 1 First Department of Internal Disease, Faculty of Medicine, Medical University, Varna, Bulgaria; 2 Faculty of Medicine, Medical University, Varna, Bulgaria

**Keywords:** acute respiratory failure, case report, human rhinovirus/enterovirus, sepsis, *Streptococcus pneumoniae*, welder

## Abstract

*Human rhinovirus/enterovirus* (*HRV/HEV*) members of the *Picornaviridae* family are traditionally associated with self-limiting upper respiratory tract infections. However, increasing evidence suggests their potential to involve the lower respiratory tract and contribute to severe pulmonary disease. *HRV/HEV* infections are frequently complicated by bacterial coinfection and the development of complicated pneumonia. This case report presents a case of a 64-year-old male welder admitted to the intensive care unit with severe right lobar pneumonia and profound hypoxemia. Microbiological investigations confirmed invasive pneumococcal disease with *Streptococcus pneumoniae* bacteremia, alongside concomitant *HRV/HEV* infection. This viral-bacterial interplay induced a dysregulated systemic inflammatory response along with coagulopathy, respiratory failure, and other multiorgan dysfunction, fulfilling Sepsis-3 criteria. Despite early initiation of targeted intravenous antibiotic therapy, the clinical course was prolonged and fluctuating. This case highlights the potential severity of *human rhinovirus/enterovirus* coinfection with invasive pathogens such as *Streptococcus pneumoniae*, contributing to complicated pneumonia and sepsis. It also underscores the increased susceptibility of welders—particularly those with additional risk factors such as smoking and inadequate vaccination—to invasive pneumococcal disease. Early recognition of viral–bacterial coinfection and prompt management of sepsis are critical to improving clinical outcomes.

## 1 Introduction

Pneumococcal pneumonia remains a leading cause of severe respiratory infection worldwide and may progress to sepsis despite appropriate therapy. Advanced age, comorbidities, and viral coinfections significantly increase the risk of adverse outcomes (1). Viral coinfections can interfere with mucociliary clearance, disrupt the epithelial barrier and create an environment suitable for bacterial superinfection (2–5).

*Human rhinovirus/enterovirus (HRV/HEV)*, taxonomically situated within the genus Enterovirus and family *Picornaviridae*, is traditionally considered as a self-limiting upper respiratory tract infection. In high-income settings, *human rhinovirus/enterovirus (HRV/HEV)* is increasingly being recognized as more than a cause of mild respiratory illness, with reports linking it to severe conditions such as acute respiratory distress syndrome and multiorgan dysfunction (6–8). In some cases, it has also been associated with extrapulmonary complications, including meningitis, encephalitis, myocarditis, and stress cardiomyopathy, which may worsen prognosis (7, 9). By contrast, data from low- and middle-income countries are still limited. This is largely due to the lack of standardized registries, with most available data coming from isolated case reports or small studies. In these settings, the burden of respiratory infections is further intensified by limited access to healthcare, a higher prevalence of comorbidities, and broader socioeconomic challenges.

The present case reflects the clinical complexity of *HRV/HEV* coinfection with *Streptococcus pneumoniae*, presenting as severe pneumonia with evidence of multiorgan involvement, and highlights the need for a better understanding of such interactions in clinical practice.

## 2 Case presentation

### 2.1 Patient information, initial assessment, and clinical findings

A 64-year-old man presented with a 4-day history of progressive dyspnea, generalized weakness, nasal congestion, myalgia, and predominantly dry cough. Because of symptoms worsening and lack of improvement with self-initiated symptomatic treatment, he was consulted by his general practitioner before admission. His medical history was notable for arterial hypertension treated with an angiotensin II receptor blocker and a calcium channel blocker. He has a smoking history of approximately 40 pack-years and works as a welder. Vaccination history revealed no prior pneumococcal, influenza, or COVID-19 immunization.

An ambulatory chest radiograph obtained before admission showed right-sided pneumonia, predominantly involving the lower and middle lung zones, with probable small pleural effusion (Figure 1A).

**Figure 1:**
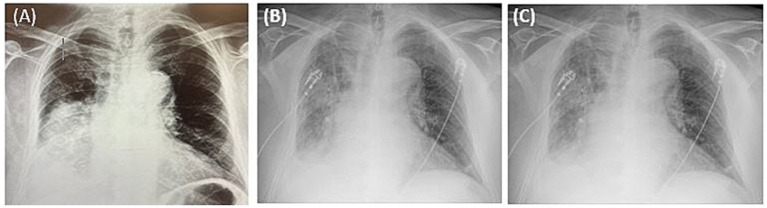
**(A)** Chest radiograph prior to hospital admission showing right-sided community-acquired pneumonia. Anteroposterior chest X-ray demonstrates a dense consolidation in the right lower and middle lung zones, consistent with lobar pneumonia. A small right-sided pleural effusion is suspected. **(B)** Follow-up chest X-ray (Day 8 post treatment initiation). Anteroposterior chest radiograph showing favorable dynamics, with partial resorption of the consolidation. **(C)** Follow-up chest X-ray (Day 15 post treatment initiation). There is significant aeration of the right lung with the resolution of the pleural effusion, though residual opacities persist in the right lower zone.

Initial laboratory testing demonstrated marked systemic inflammation, including a C-reactive protein (CRP) level of 595 mg/L (reference value <5 mg/L). Peripheral oxygen saturation on room air was 70–75%, prompting immediate referral for hospitalization.

On hospital admission (day 1), the patient was alert and oriented but in poor general condition. His temperature was 38.6 °C, respiratory rate 30 breaths/min, heart rate 110 beats/min, and blood pressure 120/80 mmHg. Oxygen saturation was 76–78% on room air. Chest examination revealed bronchial breath sounds with fine crackles over large areas of the right hemithorax, dullness to percussion, and increased vocal fremitus, consistent with extensive pulmonary consolidation. No peripheral edema or organomegaly was present.

### 2.2 Diagnostic assessment

Arterial blood gas analysis on admission showed pH 7.41, pO2 39 mmHg, pCO2 42.7 mmHg, and HCO3 27.3 mmol/L, sat. O2 74%. Hypoxemia persisted despite supplemental oxygen delivered by Venturi mask at 10–12 L/min, requiring escalation over the following days to 15 L/min via non-rebreather mask.

Initial laboratory investigations confirmed severe systemic inflammation, with leukocytosis of 18.21 × 10^9^/L and CRP 401 mg/L. Additional abnormalities suggested multiorgan involvement, including elevated bilirubin, impaired renal function, and coagulation abnormalities with increased D-dimer and fibrinogen levels (Table 1).

**Table 1:** A selection of the patient’s lab results over the course of his hospitalization.

Parameter	Reference range	Day 1 (Admission)	Day 4/6 (Peak severity)	Day 10 (Improvement)	Day 18 (Discharge)
Leukocytes (×10^9^/L)	4.0–10.0	18.2	23.5	13.6	11.8
Hemoglobin (g/L)	118–158	127	118	127	116
Platelets (×10^9^/L)	140–440	214	339	511	305
C-reactive protein (mg/L)	<5	401	277/87	53	12.6
Creatinine (μmol/L)	62–115	114	77/	—	—
Urea (mmol/L)	3.2–8.2	14.1	13.2	7.5	—
Total/Direct bilirubin (μmol/L)	<21/0–5	34/29.6	10.7/15	—	—
D-dimer (mg/L)	<0.5	2.52	—	—	—
Fibrinogen (g/L)	2.38–4.98	12.7	—	3.5	—

Computed tomography of the thorax, performed on admission, confirmed the diagnosis of right lower lobe pneumonia with involvement of small areas in the right upper lobe. Furthermore, the CT revealed an air bronchogram, a “tree-in-bud” sign, a small area of inflammation in the left lower lobe, and a small right-sided pleural effusion (less than 20 mm) not indicated for drainage (Figure 2).

**Figure 2:**
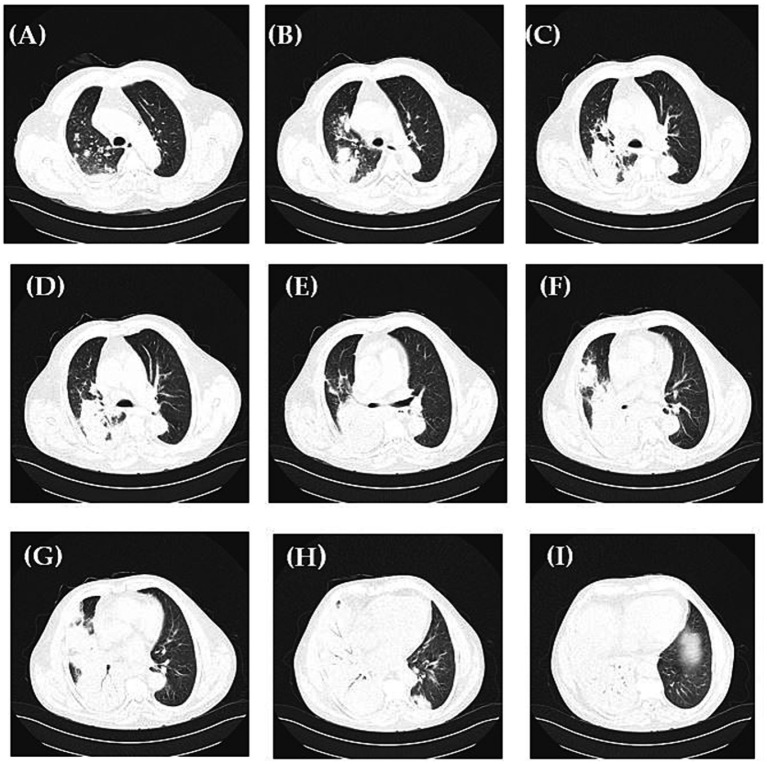
CT images of the chest obtained on admission demonstrate lobar pneumonia predominantly affecting the right lung. The scan reveals: **(A–C)** multiple inflammatory foci in the right upper/middle lobe; **(D–I)** dense consolidation with air bronchograms in the right lower lobe, alongside smaller foci of inflammation in the left lower lobe, and a small right-sided pleural effusion.

Microbiological investigation began on day 1. Reverse transcription PCR for *SARS-CoV-2* and *influenza A/B* was negative. Nasopharyngeal swab samples were analyzed on day 2, using the QIAstat-Dx Respiratory SARS-CoV-2 Panel on the QIAstat-Dx Analyzer (QIAGEN, Hilden, Germany), enabling simultaneous detection of 22 respiratory pathogens. It detected an infection with *rhinovirus/enterovirus* and was negative for the following targets: *Adenovirus, Bocavirus, Coronavirus 229E, Coronavirus HKU1, Coronavirus NL63, Coronavirus OC43, Human Metapneumovirus A/B, Influenza A subtype H1N1/2009, Influenza A subtype H1, Influenza subtype H3, Parainfluenza Virus 1, Parainfluenza Virus 2, Parainfluenza Virus 3, Parainfluenza Virus 4, Mycoplasma pneumoniae,* and *Bordetella pertussis.*

Serologic testing for acute *Mycoplasma pneumoniae* and *Chlamydia pneumoniae* infection was negative, although IgG antibodies to both pathogens were present. Urinary antigen testing for *Legionella* was negative.

Six blood culture sets were collected on days 1 and 2. Two later yielded *Streptococcus pneumoniae*, confirming invasive pneumococcal disease with bacteremia. Antibiotiv susceptibility test was performed on the *Streptococcus pneumoniae* isolated from blood. *S pneumoniae* was highly susceptible to all antibiotics tested, except for levofloxacin, where it was intermediately susceptible. Additionally, three sputum cultures obtained during the first two days of hospitalization showed no bacterial growth, and urine cultures remained sterile. On day 10, two sputum cultures grew *Candida lusitaniae*.

### 2.3 Therapeutic intervention

Empiric intravenous antibiotic therapy was started on admission with cefepime (4 g/day) plus levofloxacin (500 mg/day). Because the patient had proven infection accompanied by organ dysfunction, including severe respiratory failure (pO2/FiO2 = 185), hepatic involvement, and renal impairment, the clinical picture fulfilled Sepsis-3 criteria (10–12), with a Sequential Organ Failure Assessment score of 5. Owing to the risk of rapid deterioration, he was transferred to the intensive care unit on day 2 for close monitoring.

On day 3, the patient developed a tendency toward hypotension (95–90-85/60 mmHg) and worsening oxygenation, with progressive decline in the PaO2/FiO2 ratio to 75. In the context of severe bacterial pneumonia, evolving sepsis, and newly confirmed *HRV/HEV* coinfection, cefepime was replaced with meropenem (4 g/day). Preliminary blood and sputum culture results were still negative at that time. Because bronchoconstriction was also present, systemic corticosteroid therapy (methylprednisolone 40mg) was added on day 4. Supportive treatment included high-flow oxygen supplementation, intravenous fluids, thromboprophylaxis with nadroparin, administration of vasoactive agents (Dopamine), and symptomatic therapy. No specific antiviral therapy was available for *HRV/HEV* infection.

### 2.4 Clinical course and follow-up during hospitalization

During the ICU stay, respiratory failure initially worsened, requiring escalation of oxygen therapy to 15 L/min via non-rebreather mask. The patient subsequently developed a productive cough with yellow purulent sputum. Overall, the course was prolonged and fluctuating.

Renal function and bilirubin levels normalized relatively early after initiation of treatment. However, inflammatory markers showed a dissociated pattern. On day 6, the leukocyte count increased further to 23.5 × 10^9^/L, despite a marked decline in CRP to 87 mg/L. At that stage, respiratory failure remained severe and high-flow oxygen supplementation was still required as well as administration of vasoactive agents.

On day 7, one blood culture yielded *Micrococcus luteus*, which was interpreted by the microbiology laboratory as probable contamination. Given the patient’s overall clinical trajectory and the absence of radiological evidence of progression, antimicrobial therapy was not changed. Follow-up chest radiography on day 8 demonstrated partial radiologic improvement with regression of the consolidation (Figure 1B).

On day 10, two sputum cultures grew *Candida lusitaniae*, and fluconazole was added according to the antibiogram results. Thereafter, the patient’s condition improved progressively. CRP decreased further to 53 mg/L by day 10. Repeat arterial blood gas analysis showed improved oxygenation with reduced oxygen requirements. A follow-up chest radiograph obtained before discharge demonstrated almost complete resolution of the consolidation and pleural effusion, with only residual right lower zone opacities persisting (Figure 1C).

### 2.5 Treatment, outcome, and post-discharge follow-up

The patient received broad-spectrum antimicrobial therapy with cefepime followed by escalation to meropenem and levofloxacin. Adjunctive treatment included methylprednisolone, thromboprophylaxis, intravenous fluids, administration of vasoactive agents, and high-flow oxygen supplementation. Fluconazole was initiated following identification of *Candida lusitaniae* in two sputum cultures. Yeast forms are seen on Gram stain and *Candida* is cultured in sputum cultures. No targeted antiviral therapy was available for *HRV/HEV* infection; management remained supportive.

The clinical course was initially severe but gradually improved, with resolution of fever, normalization of renal and hepatic function, and near-complete radiological recovery at discharge. The patient was discharged on day 18 in a good general condition, afebrile, hemodynamically stable, and without the need for supplemental oxygen. Renal and liver function had normalized, and radiologic findings had markedly improved. He was advised to stop smoking and to receive pneumococcal and COVID-19 vaccination.

At 15-day and 30-day follow-up visits, physical examination was unremarkable, and laboratory tests and blood gas analyses were within normal limits. However, the patient continued to report fatigue, reduced exercise tolerance, and a subjective inability to return to work.

## 3 Timeline of clinical course

A chronological summary of the patient’s clinical course is presented in Table 2.

**Table 2:** Timeline of clinical course.

Day	Clinical events & findings	Diagnostics	Interventions	Outcome/response
Day −0 to 4 before admission	Onset of dyspnea, cough, myalgia, weakness; worsening symptoms	GP evaluation; chest X-ray shows right-sided pneumonia; CRP 595 mg/L; SpO₂ 70–75% (room air)	Symptomatic treatment (self-initiated)	No improvement → referral to hospital
Day 1 (Admission)	Fever (38.6 °C), tachypnea, severe hypoxemia	ABG: severe hypoxemia – pO2/FiO2 = 185; leukocytosis; CRP 401 mg/L; CT: right lobar pneumonia, pleural effusion	Oxygen (Venturi mask 10–12 L/min); cefepime + levofloxacin started	Persistent hypoxemia
Day 2	Clinical deterioration risk	Multiplex PCR positive for *HRV/HEV*; blood/sputum/urine cultures taken; PaO₂/FiO₂ = 85–75	Transfer to ICU; Oxygen increased to 15 L/min (non-rebreather mask)	Close monitoring initiated
Day 3	Worsening respiratory failure; hypotension	PaO₂/FiO₂ declining to 75	Antibiotics escalated (meropenem); administration of vasoactive agents required	Continued instability
Days 4–5	Severe respiratory failure persists	Ongoing lab monitoring	Corticosteroids added; administration of vasoactive agents	Gradual stabilization begins
Day 6	Leukocytosis peak (23.5 × 10^9^/L); CRP decreasing	Lab dissociation noted	Continued therapy	Suspicion of inflammatory response vs. superinfection
Day 7–8	Clinical monitoring continues	Blood culture: *Micrococcus luteus* (contaminant), and *Str, pneumoniae* – positive; CXR: partial resolution of consolidations	No therapy change	Stable condition, radiological improvement
Day 10	Further clinical improvement	CRP ↓ to 53 mg/L; sputum: *Candida lusitaniae*	Fluconazole initiated for a total of 10 days	Improved oxygenation with oxygen reduction
Days 12–15	Progressive recovery	Improved ABG	Gradual oxygen reduction Deescalation of antibiotics/Levofloxacin on day 14.; Meronem monotherapy for another 2 days/	Stabilization
Day 18 (Discharge)	Clinically stable, no oxygen needed	Resolution on imaging	Discharged with recommendations	Good recovery
Follow-up (15 & 30 days)	Persistent fatigue, reduced exercise tolerance	Normal labs and ABG	Smoking cessation + vaccination advised	No relapse

## 4 Patient perspective

The patient reported persistent fatigue and reduced exercise tolerance following discharge, which delayed return to work. He expressed satisfaction with the care received and reported gradual improvement over time.

## 5 Discussion

The role of *human rhinovirus/enterovirus (HRV/HEV)* as a clinically significant pathogen has been increasingly recognized with the widespread use of sensitive diagnostic methods such as multiplex PCR. Although typically associated with mild upper respiratory tract infections, *HRV/HEV* is now implicated in severe lower respiratory tract disease, including acute respiratory distress syndrome (ARDS) (6, 7) and multiorgan dysfunction (8).

*HRV/HEV* infections can, in rare cases, lead to severe complications such as myocarditis (7), meningitis, and encephalitis (8, 9). Moreover, viral–bacterial coinfection is common in severe community-acquired pneumonia, with *HRV/HEV* detected in up to 25–30% of severe cases (2). Additionally, *adenovirus/RV* and *HEV/HRV* were the two most incident concomitant viral pairs (1.43 and 0.83%), while *HRV* and *Str. pneumoniae* was one of the top 3 viral-bacterial pairs (13). Coinfection with pathogens such as *Str. pneumoniae* is associated with worse clinical outcomes, including prolonged hospitalization and increased mortality (14–17).

In the present case, invasive pneumococcal disease with bacteremia was accompanied by *HRV/HEV* coinfection, leading to rapid progression to sepsis. Occupational exposure as a welder represents an important contributing factor. Epidemiological investigations show significantly increased risk for welders developing severe pneumococcal pneumonia. On the one hand, an inhalation of metallic vapors (primarily iron particles) induces oxidative stress and impairs pulmonary macrophage function, thereby compromising local immune defense and facilitating bacterial invasion (18). On the other hand, the infection with *HRV/HEV* induces injury to the respiratory epithelium and impairs mucociliary clearance, allowing for adherence to and invasion of *Str. pneumonia*. The precise pathogenic mechanisms of *HRV/HEV* infection remain not fully understood; however, it is likely that they involve a combination of direct viral damage to respiratory epithelial cells and a dysregulated innate inflammatory response of the host driven by cytokines, including the stimulation of IFN-*β*, IFN-*γ*, IL-1, IL-6, and IL-8 expression, partly mediated through an NF-κB-dependent transcriptional activation pathway (3, 4, 19).

Clinically, this case was characterized by rapid onset of sepsis according to Sepsis-3 criteria, with respiratory, renal, and hepatic dysfunction. The initial Sequential Organ Failure Assessment (SOFA) score of ≥5 indicated a high risk of mortality and corresponded with the severe clinical course observed in populations or cohorts with similar scores (12).

An important observation was the dissociation between inflammatory markers, with sharp increase in leukocytes despite declining CRP levels and clinical improvement. This pattern may reflect delayed inflammatory response, corticosteroid effects (less than 24 h of application), or leukocyte mobilization rather than ongoing infection. The decision to continue antimicrobial therapy based on overall clinical assessment rather than isolated laboratory values proved appropriate.

This case also emphasizes the importance of microbiological interpretation. The detection of *Micrococcus luteus* was considered contamination, and no unnecessary escalation of therapy was undertaken. This highlights the need for careful correlation of laboratory findings with clinical context.

Several limitations should be acknowledged. First, it was not possible to determine the temporal relationship between viral and bacterial infection. Second, viral load quantification was not performed. Third, BAL specimens, which are generally considered superior to URT samples for both multiplex PCR and bacterial cultures, were not obtained. Finally, delayed availability of microbiological results necessitated treatment decisions.

## 6 Conclusion

This case highlights the potential severity of *HRV/HEV* coinfection in combination with invasive pathogens such as *Str. pneumoniae*, contributing to complicated pneumonia and sepsis. The resulting dysregulated inflammatory response may lead to rapid multiorgan dysfunction despite appropriate therapy.

It also underscores increased susceptibility among welders, particularly those with smoking history and inadequate vaccination. Early identification of viral–bacterial coinfection, careful clinical monitoring, and preventive strategies—including pneumococcal vaccination—are essential to improve outcomes in high-risk populations.

## Data Availability

The raw data supporting the conclusions of this article will be made available by the authors, without undue reservation.
